# The influence of the type of breastfeeding on middle ear conditions in infants

**DOI:** 10.1590/S1808-86942012000100002

**Published:** 2015-10-20

**Authors:** Michele Vargas Garcia, Marisa Frasson de Azevedo, José Ricardo Gurgel Testa, Cyntia Barbosa Laureano Luiz

**Affiliations:** a PhD student in Communication Disorders - UNIFESP/EPM, Speech and Hearing Therapist.; b PhD, Adjunct Professor - Federal University of São Paulo - School of Medicine (EPM-UNIFESP); c PhD, Adjunct Professor - UNIFESP/EPM.; d Audiology Student - UNIFESP/ EPM, Speech and Hearing Therapist. Universidade Federal de São Paulo- UNIFESP.

**Keywords:** breast feeding, ear, middle, neonetal screening

## Abstract

Infants should be submitted to hearing screening upon birth, and for the results to be complete, it is necessary to assess middle ear conditions.

**Objective:**

To check whether the type of breastfeeding in infants between zero and four months can impact middle ear conditions my means an ENT assessment and acoustic immittance comparing neonates who were submitted to hearing screening with those who failed it.

**Materials and Methods:**

Otoacoustic emissions (OAE) was carried out in 60 infants between zero and four months. They were distributed in two groups; group I had the infants with OAE and those infants in group II did not have OAE. They were submitted to tympanometry with a 1000 Hz test tone and ENT assessment.

**Results:**

Bottle fed infants or those who were fed in a mixed way had more changes to their audiometry and ENT assessment, with a statistically significant difference. The breastfed infants had a higher occasion of normal tympanometries and normal otorhinolaryngological assessment, with statistically significant difference.

**Conclusion:**

We then concluded that those breastfed implants had less ENT changes and as well as less acoustic immittance change, thus enabling OAEs. Breastfeeding alone can be considered a protection factor against middle ear changes.

## INTRODUCTION

All infants must be submitted to hearing screening soon after birth; and in order to have reliable results, it is necessary to assess the infant's middle ear conditions.

Middle ear conditions can interfere in otoacoustic emissions, because the click stimulus must go through the external and middle ears in order to reach the cochlea, and the emissions response also goes through the middle and external ears until they are captured in the external ear canal. Acoustic immittance measures contribute with information about middle ear ossicle mobility and it also says something about the hearing pathway at this level, and the tympanometric curves are classified by Jerger. They are very much used with infants in clinical practice because they represent an objective evaluation providing the tympanometric curves and acoustic reflexes. Middle ear changes can be influenced by the type of breastfeeding the infant is receiving. When infants are breastfed they may be better protected as far as their health is concerned^1^, especially against infections.

Breastfeeding advantages to neonates are unquestionable. When exclusive breastfeeding is not possible, it is necessary to introduce artificial milk to replace nutrients, abiding to the child's neurophysiological development.

The neonate's hearing assessment is carried out by means of Otoacoustic Emissions (OAE), which evaluate cochlear function (outer hair cells). In order for such assessment to be trustworthy, it is important to assess the middle ear, especially in those cases when the OAEs fail. Thus, middle ear diseases may impact otoacoustic emission recording and may delay hearing diagnoses. These changes can be diagnosed by means of an ENT evaluation and acoustic immittance measures.

If the infant is breastfed, he/she will be better protected as far as health is concerned, and without middle ear changes, they yield better results upon hearing assessment. Breastfed infants are better protected against upper airway infections^2^.

Howie et al.^3^ and Goldman et al.^4^ reported that breast milk protects infants against infections, and mention the protection provided by the mother's milk colostrum.

This study's justification is based on the need to know the type of breastfeeding the infants are receiving and the influence of middle ear health conditions. Thus, in this study we aimed at investigating the type of breastfeeding infants between zero and four months of age were receiving and the influence of middle ear conditions in the otorhinolaryngological assessment and acoustic immittance, comparing neonates who passed neonatal hearing screening with those who did not.

## METHODS

This study was carried out after being approved by the Ethics in Research Committee with the following protocol #: 0723/06.

Following ethical principles of studies with human beings, the parents and/or guardians agreed with the participation of their children in the present study and signed an informed consent form.

The sample was made up of 60 infants, from both genders, with ages ranging between zero and four months, born in São Paulo, who were submitted to neonatal screening. The neonates were distributed in two groups.

• Group I: Thirty infants with transient stimulus evoked otoacoustic emissions, 18 females and 12 males.
• Group II: thirty infants without transient stimulus evoked otoacoustic emissions, 9 females and 21 males.

In order to make up the groups, the infants had to be between zero and four months, with or without risk indicators for hearing impairment. We took off the study all infants with external ear canal malformation, because it would make it impossible to carry out the assessments proposed by the present investigation.

All the children were submitted to otorhinolaryngological exam and immittance measures in the same date the otoacoustic emissions study was carried out. The study was double blinded, in other words, the examiners were not aware of the results from the other tests the infants had been submitted to. The parameters considered in the present study were the following:

### 1. Recording and analysis of the Transient Evoked Otoacoustic Emissions (TEOAE)

The infants were submitted to recording and analysis of their transient evoked otoacoustic emissions (TEOAE) by means of click stimulus, with a stimulus intensity of 75-83dBpeSPL. We considered TEOAE present when the signal-to-noise ratio per frequency band was ≥ 3 dB for 1500Hz, and ≥ 6 dB for 2000Hz, 3000 and 4000Hz; the general reproducibility considered was ≥ 50%, and the probe stability ≥70%^5^, ^6^. The transient evoked otoacoustic emissions were studied with the infants inside an acoustically treated booth. The equipment utilized was the *ILO 96-Otoacoustic Emissions Analyzer,* connected to a microcomputer, using the *“Quickscreener”* software.

The groups were formed based on the TEOAE results.

### 2. Otolaryngological evaluation

The infants were assessed by an ENT physician, who did an otoscopic exam to check the external ear canal and the tympanic membrane. For this study, the tympanic membrane was classified as normal or altered (retracted, hyperemic, opaque, perforated, bulged). The physician in charge of the assessment had over fifteen years of experience with newborns and was not aware of which group the infant belonged to.

### 3. Acoustic immittance measures

The infants were submitted to tympanometry by means of the Middle Ear Analyzer: *Impedance Audiometer* - AT235h- *Interacoustics.* The tympanometric curve was carried out with a 1000Hz test tone.

Tympanometric curves were classified in: Type A curve - admittance single peak between −150 and + 100 daPa and volume between 0.2 and 1.8ml; Type C curve - the admittance curve shifted towards negative pressure; type-D curve in double peak; Asymmetrical curve - peak in high positive pressure; Inverted curve - an inverted curve in relation to the normal curve and type B flat curve with and without admittance peak^7^, ^8^, ^9^.

The contralateral acoustic reflexes were investigated in the frequencies of 500; 1,000; 2,000 and 4,000 Hz and the ipsilateral ones in 1,000 and 2,000 Hz, utilizing the same aforementioned equipment.

The examiner did not know to which group the infant belonged.

### 4. Anamnesis

The infant's mother or guardian was asked about the type of feeding to which the infant was submitted, and the options were as follows: breastfeeding only, artificial feeding from a bottle only or mixed (breastfeeding and bottle).

The results from this study were statistically analyzed by means of the equality test of two proportions.

## RESULTS

The results will be presented by groups. GI: 30 infants with TEOAEs and GII with 30 infants without TEOAE.

First, we analyzed the findings from the otolaryngological evaluation, considering the tympanic membrane conditions (Table 1).

**Table 1:** Results from the Otorhinolaryngological evaluation in groups I (emissions present) and II (emissions absent).

Otorhinolaryngological Assessment	Normal	Altered	Total number of infants
Group I	18 (60%)	12 (40%)	30 (100%)
Group II	11 (36.6%)	19 (63.4%)	30 (100%)
Total	29 (48.4%)	31 (51.6%)	60 (100%)
*p*-value	0.071#	0.071#	

* significant *p*-value < 0.005 (5%).

Group I: infants with otoacoustic emissions; Group II: infants without otoacoustic emissions.

Considering the otorhinolaryngological evaluation of the infants from the groups, there was a statistical trend from group I to have more infants with normal otoscopic evaluation and group II with more infants with changes found upon otoscopy.

On Table 2 one can see the occurence of otorhinolaryngological changes (bilateral or unilateral).

**Table 2:** Unilateral or bilateral otorhinolaryngological changes in Groups I and II.

Otorhinolaryngological change	Bilateral	Unilateral	Total number of ears
Group I	8 (66.6%)	4 (33.4%)	12 (100%)
Group II	17 (89.5%)	2 (10.5%)	19 (100%)
Total	25 (80.6%)	6 (19.4%)	31 (100%)
*p*-value	0.117	0.117	

* significant *p*-value < 0.005 (5%).

Group I: infants with otoacoustic emissions; Group II: infants without otoacoustic emissions.

There were no differences between the groups in relation to the occurrence of unilateral or bilateral otorhinolaryngological changes.

Otorhinolaryngological evolution in relation to the type of otoscopic change are depicted on Table 3.

**Table 3:** Type of change found upon otoscopy (number of ears) obtained from the groups studied.

TM	Normal	Retracted	Hyperemic	Opaque	Total Number of ears
G I	40 (66.6%)	1 (1.7%)	1 (1.7%)	18 (30%)	60 (100%)
G II	24 (40%)	2 (3.3%)	0	34 (56.7%)	60 (100%)
Total	64	3	1	52	120 (100%)
*p*-valor	0.003*	0.559	0.315	0.003*	

Group I: infants with otoacoustic emissions, Group II: infants without otoacoustic emissions

* significant *p*-value < 0.005 (5%).

The type of otoscopic change found was statistically different between the groups. In Group I there was a higher occurrence of normal otoscopies, and in Group II a higher occurrence of opaque tympanic membranes.

Tympanometry was carried out with the test tone of 1,000Hz and the results are depicted on Table 4.

**Table 4:** Results from the test tone tympanometry at 1,000 Hz from the study groups.

Tympanometry at 1,000Hz	Normal bilateral	Changed bilateral	Changed Unilateral	Total number of infants
Group I	22 (73%)	1 (4%)	7 (23%)	30 (100%)
Group II	6 (20%)	23 (76%)	1 (4%)	30 (100%)
Total number of infants	28 (46%)	24 (40%)	8 (14%)	60 (100%)
*p*-value	<0.001*	<0.001*	0.023*	

* significant *p*-value < 0.005 (5%.). Group I: infants with otoacoustic emissions; Group II: infants without otoacoustic emissions.

The tympanometry carried out with the 1,000Hz test tone showed statistical differences between the groups. Group I had a higher occurrence of infants with normal bilateral tympanometric curves. In Group II we found a higher percentage of infants with bilaterally changed tympanometric curves. When there were GI changes, they occurred predominantly in GI.

The types of curves obtained from each group are depicted on Table 5.

**Table 5:** Types of tympanometric curves with the 1,000Hz test tone obtained from Groups I and II.

Types of curves	"A"	"D”	"I"	"B"	"C"	Total number of ears
Group I	36 (60%)	7 (11,6%)	8 (13,4%)	3 (5%)	6 (10%)	60 (100%)
Group II	12 (20%)	1 (2%)	0	42 (70%)	5 (8%)	60 (100%)
Total	48 (40%)	8 (6.6%)	8 (6.6%)	45 (37.5%)	11 (9.3%)	120 (100%)
*p*-value	<0.001*	0.028*	0.003*	<0.001*	0.752	

* significant *p*-value < 0.005 (5%). Group I: infants with otoacoustic emissions, Group II: infants without otoacoustic emissions. Types of tympanometric curves: “A” normal, “D” double peak, “I” inverted,”B” flat without a peak, “C” peak shifted to negative pressure.

There was a statistically significant difference between the tympanometric curves found with the use of the 1,000Hz test tone. The Type A tympanometric curve was more frequent in group I, as well as the double peak curve and the I type. the type B tympanometric curve happened more frequently in Group II.

As to the feeding received, the results are depicted on Table 6.

**Table 6:** Type of feeding received by the infants in the groups studied.

Feeding	Breastfeeding only	Bottle only	Mixed	Total
Group I	21 (70%)	5 (16,7%)	4 (13,3%)	30 (100%)
Group II	10 (33.3%)	11 (36.7%)	9 (30%)	30 (100%)
Total	31 (51.6%)	16 (27%)	13 (21.4%)	60 (100%)
*p*-value	0.004*	0.080#	0.117	

* significant *p*-valor < 0,005 (5%). Group I: infants with otoacoustic emissions; Group II: infants without otoacoustic emissions.

It was noticed that breastfed infants are more present in Group I, in other words, had transient otoacoustic emissions.

The statistical analysis showed that the infants who received breastfeeding had less changes to their tympanic membrane and those fed by bottle or mixed feeding had a higher number of changes seen upon otorhinolaryngological evaluation.

We noticed that breastfed infants had more tympanometric curves within normal ranges than mixed-feeding or bottle-feeding-only infants had more altered tympanometric curves.

## DISCUSSION

When we are born, human beings have only the reflex-type of hearing, which is inhibited when the learning process starts, with new responses to sound depending on our hearing experiences. The importance of submitting newborns to otoacoustic emissions (OAEs) is based on the fact that it indicates whether or not the peripheral auditory pathway is intact, more specifically the cochlear outer hair cells. When OAEs are present, the peripheral hearing is intact (acuity up to 30 dB HL)^10^. Parrado^11^ and Weber & Diefendorf^12^ point to the use of OAEs for the auditory assessment of infants, for it is a fast and objective test.

Infants who fail their OAEs must be examined by an ENT physician. It is necessary that the examiner have experience with infants because of the difficulty in assessing their tympanic membranes, having in mind the narrowness of the external ear canal.

OAEs were easily measured in the two groups studied, and when infant failed OAEs, the test was repeated for result confirmation purposes. This finding is in agreement with the study^13^, who concluded that the TOAEs were easily measured.

There was a statistically significant association between the otorhinolaryngological examination (in both ears) with the otoacoustic emissions, considering the correlation of the missing otoacoustic emissions with the altered medical evaluation and the emissions present with a normal medical examination. Table 1 showed that 60% from Group I with TEOAE, have normal ENT exam and 63.4% from Group II without TEOAE. These findings are in agreement with the citations from Trine et al.^14^ and Owen et al.^15^ who reported a good correlation between OAEs and the ENT exam. Such assessment, when carried out by an otorhinolaryngologist, provides the audiologist more reliability upon examining the results from otoacoustic emissions.

These findings are also in agreement with those from Silva^16^, who reported excellent correlation between TEOAEs and the ENT examination to differentiate conduction diseases from their cochlear counterparts. That conductive hearing disorders also influence OAE results^17^.

Carvallo et al.^10^ stress that middle ear diseases can impact the collection of Otoacoustic Emissions, and findings from the current study are in agreement with them.

The analysis of the methods employed to do the tympanometry is paramount for the audiologist to be certain concerning results, since the difference in the tympanometric curve impacts the type of hearing loss the infant may present with. The middle ear immittance study provides a large number of practical diagnostic practices, such as, for example, information on the functional integrity of the tympanic-ossicular chain.

There was a statistically significant correlation for the 1,000Hz test tone and otoacoustic emissions both for infants with changes in their evaluations as for those who had normal results. Table 4 showed that 73% of the infants with normal bilateral tympanometry are in group I and 76% of infants with bilateral changed tympanometry are in Group II. Owens et al.^15^ and Calandruccio et al.^18^ carried out a study with infants using OAEs and tympanometry with the 1,000 Hz test tone, and found good correlations between the tests, and the same was found in our sample. Otoacoustic emissions are very sensitive to middle ear changes, which is in agreement with the present study^19^, ^20^.

Zapala^21^ and Koivunem et al.^22^ have correlated the OAEs with multiple frequency tympanometries in neonates in order to look for conductive hearing diseases. In the present study, there is an agreement between the findings of the above-mentioned authors, because they report greater reliability concerning the 1,000 Hz tone test upon tympanometry when done together with OAEs. Sutton et al.^23^, McKinley et al.^24^ Margolis^25^ believe the correlation between OAEs and 1,000Hz tympanometry to be good.

The findings have shown that 8% of Group II infants (without OAEs) had a type “C” tympanometric curve (Table 5), with the 1,000 Hz test tone, which indicates negative pressure in the middle ear. This finding is in agreement with Marshall et al.^26^ who reported that small amounts of negative pressure in the middle ear could impact OAE spectrum and amplitude.

Mother's milk protects the infant from infections^1^, ^3^, ^4^ and the authors mention the protection provided by the colostrum present in the mother's milk. The benefits of maternal milk are well known, and campaigns are often put in motion in order to have the mother's milk increasingly valued by the mothers. Among the benefits to the infant's health, brought about by the mother's milk, we have the protection against infections. Breastfeeding can also provide a better infant position to feed, since the infant's head is supported on the mother's arm at a proper height, more horizontally, preventing the milk from going to the auditory tube. When the infant is fed with artificial milk in the bottle, besides missing on the benefits of the maternal milk, the baby has greater risk of being fed in a more vertical position, which may cause milk to run down to the auditory tube and into the middle ear, thus causing infection.

The mothers of these infants were asked about the type of feeding received by the infant (breastfeeding only, mixed and bottle with artificial milk only) and received instructions as to the position of the baby for feeding. The goal was to check whether or not the infants fed maternal milk had more middle ear protection when compared to those fed through a bottle.

The group with OAEs had a statistically significant number of infants who were breastfed (70%) and Group II, with more infants being bottle fed - 36.7% of the group (Table 6). Children with normal cochlear function had a predominance of having been breastfed.

In our sample, health protection was noticed in the middle ear of infants by means of recording OAE, tympanometry and otorhinolaryngological evaluation (Tables 7 and 8). The statistical analysis was carried out by comparing the infants in their groups. In Group I, 70% of the infants from the group were breastfed only, and 13.3% received mixed feeding with breast milk and artificial milk and 30% received mixed feeding. Through statistical data, we can notice that breastfeeding alone brings benefits to the infants when one considers middle ear conditions, since Group I infants were the ones who had OAEs, with normal cochlear function and without middle ear changes.

**Table 7:** Correlation between feeding type and otolaryngological exam.

ENT exam(TM)		Changed		Normal		Total		*p*-value
		N	%	N	%	N	%	
Bottle only	G1	3	16%	7	54%	10	31%	
	G2	16	84%	6	46%	22	69%	0,023*
	Total	19	59%	13	41%	32	100%	
Breastfeeding only	G1	15	60%	27	73%	42	68%	
	G2	10	40%	10	27%	20	32%	0,028*
	Total	25	40%	37	60%	62	100%	
	G1	2	14%	6	50%	8	31%	
Mixed	G2	12	86%	6	50%	18	69%	0,049*
		14	54%	12	46%	26	100%

**Table 8:** Correlation between tympanometry with the 1,000Hz test tone and the type of feeding.

Tymp (1000 Hz)		Changed		Normal		Total		*p*-value
		N	%	N	%	N	%	
Bottle only	G1	3	14%	7	64%	10	31%	
	G2	18	86%	4	36%	22	69%	0,004*
	Total	21	66%	11	34%	32	100%	
Breastfeeding only	G1	4	22%	38	86%	42	68%	
	G2	14	78%	6	14%	20	32%	<0,001*
	Total	18	29%	44	71%	62	100%	
	G1	2	12%	6	67%	8	31%	
Mixed	G2	15	88%	3	33%	18	69%	0,004*
		17	65%	9	35%	26	100%	

From the total of 60 infants, 29 (48.3%) had their breastfeeding-only diet broken for some reason. This interruption in breastfeeding may have influenced middle ear conditions, enabling a greater rate of OAE failures and otorhinolaryngological failures. Determining factors for early interruption of breastfeeding have been investigated and, there are many factors which trigger this interruption, such as mother's educational level and her insertion in the work market^27^.

The percentage of breastfeeding only in the present study (51.7%) (Table 6) was higher than what was found in the study led by Bueno et al. (2002) who studied 450 infants between zero and one year of age, with the goal of checking the time of breastfeeding only after the introduction of another milk in the child's diet. Of the entire sample, 54 (12%) infants were breastfed exclusively until they turned two months of age. After such age, the infants received another type of feeding.

These findings match those from some authors, because they noticed that breastfed infants have less middle ear disorders; and when they are bottle-fed they have these disorders, and they may recur 28, 29.

In this study, infants were given a bottle with milk powder recommended for infant age in the study, which is consistent with Pernetta^30^ Fomon^31^ and which relate to the need for assessments in infants correct introduction of food to replace breastfeeding.

In this sample, the infants were between zero and four months of age and during this period the mother's milk alone is indicated and this is mentioned in the literature^32^. Thus, the ideal situation for all infants in the sample should be breastfeeding only; nonetheless, of the 60 infants from both groups in this study, 29 (48.3%) were not breastfed exclusively; and of these, 20 (69%) failed OAEs because of middle ear disorders. When the infant is fed for a short time with the mother's milk exclusively, it lowers the individual's immunity, rendering the infant susceptible to infections and other diseases^33^.

The results found are in agreement with the findings from Howie et al.^3^ and Strassburger^32^, which advocate that breastfed infants have more protection to their health, especially against infections. The breastfed infants in the study had less middle ear changes identified by ENT exam and by acoustic immittance (Tables 7 and 8).

Considering the 60 infants from both study groups, 51.7% of the infants (Table 6) were breastfed, which is in agreement with the data reported by the Ministry of Health in 2001 for the age range studied. In Brazil, regardless of the good results achieved by breastfeeding campaigns, this breastfeeding duration is still short of its goals^34^.

## CONCLUSION

Based on our findings, we concluded that those infants who were breastfed have less changes upon ENT examination and in acoustic immittance measures, thus having OAEs.
